# The expression of microRNA 574-3p as a predictor of postoperative outcome in patients with esophageal squamous cell carcinoma

**DOI:** 10.1186/s12957-016-0985-3

**Published:** 2016-08-26

**Authors:** Tomoyuki Okumura, Hirohumi Kojima, Takeshi Miwa, Shinichi Sekine, Isaya Hashimoto, Shozo Hojo, Takuya Nagata, Yutaka Shimada

**Affiliations:** 1 Department of Surgery and Science, Graduate School of Medicine and Pharmaceutical Sciences, University of Toyama, 2630 Sugitani, Toyama, 930-0194 Japan; 2 Department of Nanobio Drug Discovery, Graduate School of Pharmaceutical Sciences, Kyoto University, Kyoto, Japan

**Keywords:** Esophageal cancer, Squamous cell carcinoma, miRNA, Prognostic marker, Surgery

## Abstract

**Background:**

Despite advances in radical esophagectomies and adjuvant therapy, the postoperative prognosis in esophageal squamous cell carcinoma (ESCC) patients remains poor. The aim of this study was to identify a molecular signature to predict postoperative favorable outcomes in patients with ESCC.

**Methods:**

As a training data set, total RNA was extracted from formalin-fixed paraffin-embedded samples of surgically removed specimens from 19 ESCC patients who underwent curative esophagectomy. The expression of microRNA (miRNA) was detected using a miRNA oligo chip on which 885 genes were mounted. As a validation data set, we obtained frozen samples of surgically resected tumors from 12 independent ESCC patients and the expression of miR-574-3p was detected by quantitative real-time PCR.

**Results:**

Our microarray analysis in the training set patients identified three miRNAs (miR-574-3p, miR-106b, and miR-1303) and five miRNAs (miR-1203, miR-1909, miR-204, miR-371-3p, miR-886-3p) which were differentially expressed between the patients with (*n* = 14) and without (*n* = 5) postoperative tumor relapse (*p* < 0.01 and *p* < 0.05, respectively). Higher expression of miR-574-3p, which showed the most significant association with non-relapse (*p* = 0.001), was associated with favorable overall survival (*p* = 0.016). Real-time PCR experiments on the validation set patients confirmed that higher expression of miR-574-3p was associated with non-tumor relapse (*p* = 0.029) and better overall survival (*p* = 0.004).

**Conclusions:**

Our results suggest that the aberrant expression of the miRNAs identified in this study plays key roles in the progression of ESCC. miR-574-3p was suggested to have a tumor suppressor effect, and thus, to be a predictor of postoperative outcome in patients with ESCC.

**Electronic supplementary material:**

The online version of this article (doi:10.1186/s12957-016-0985-3) contains supplementary material, which is available to authorized users.

## Background

Recent progress in radical esophagectomies and perioperative adjuvant therapy has facilitated an improved postoperative prognosis, even in patients with advanced esophageal squamous cell carcinoma (ESCC) [1]. However, many of the patients still exhibit postoperative tumor recurrence with a 5-year progression-free survival rate of about 40 % [2, 3]. Therefore, innovative strategies to predict early postoperative tumor relapse may provide us with a more accurate diagnosis to determine operative indications.

MicroRNAs (miRNAs) are small, single-stranded, non-coding RNAs that play a key role in the initiation and progression of cancer through the post-transcriptional regulation of gene expression [4]. They have also been reported to be potential biomarkers for therapeutic effects and prognoses in cancer patients [5].

In the present study, we investigated the expression profiles of miRNAs in surgically removed ESCC specimens to identify relapse-associated miRNAs.

## Methods

### Patients and surgical specimens

We selected eighty-three consecutive ESCC patients who underwent curative surgery in Toyama University Hospital between 1991 and 2008, excluding 16 cases who died of other causes within 5 years after surgery. Then, we obtained formalin-fixed paraffin-embedded (FFPE) samples of the tumors and their normal counterparts, with adequate tissue size from 36 cases as a training data set. In addition, we obtained frozen samples of surgically resected tumors and their normal counterparts, which had been kept at −80 °C, from 12 independent ESCC patients who received curative surgery in our hospital between 2008 and 2010 to use as a validation data set. The observation period was calculated from the date of surgery until the patient succumbed to the disease or the last follow-up contact. All cases were staged according to the International Union Against Cancer TNM Classification 7th edition [6].

### RNA extraction from FFPE specimens

Sections (10 μm) were prepared from each FFPE specimen. Paraffin was removed by xylene treatment, and the tissues were then washed with ethanol twice to remove the xylene. The tissues were treated with proteinase K at 37 °C overnight. Following centrifugation, the supernatant was processed with a silica-based spin column (Toray Industries, Japan) in order to obtain purified total RNA. The degrees of RNA cross-linking and RNA degradation were analyzed by electrophoresis using an Agilent 2100 Bioanalyzer (Agilent Technologies, Santa Clara, CA, USA).

### miRNA assays using microarray

miRNA profiling was examined using a Toray 3D-Gene® miRNA oligo chip (Toray Industries), on which 885 genes were mounted. The detailed procedure for this experiment has been described previously [7]. The expression level of each miRNA was normalized using the median signal strength for the entire gene in each chip.

### RNA extraction from frozen specimens and quantitative RT-PCR analysis for microRNA s

Frozen specimens were obtained from surgically removed esophagus with Lugol’s solution staining. Tumor samples were cut from main part of the tumor, and normal tissues from mucosal epithelia with positive Lugol staining, more than 5 cm distant from the tumor edge. The sections were also confirmed by a board-certified pathologist. Total RNA was extracted from frozen specimens using the TRIzol Reagent (Invitrogen, Carlsbad, CA, USA) according to a standard protocol. cDNA was prepared from total RNA samples using the Taq Man microRNA reverse transcription kit on the ABI Prism 7000 real-time PCR system, according to the manufacturer’s instructions (Applied Biosystems). Predesigned Taq Man microRNA assays for hsa-miR-574-3p (Assay ID 002349) and RNU6B (Assay ID 001093) were purchased from Applied Biosystems. qRT-PCR was performed using a Taq Man universal PCR master mix, according to the manufacturer’s protocol (Applied Biosystems). The microRNA quantities were analyzed in duplicate and normalized against U6B as an internal control. The tumor-to-normal ratio (T/N ratio) was calculated based on the miRNA expression levels in each tumor and the corresponding normal counterpart.

### Statistical analysis

All analyses were carried out with JMP 9.0 software (SAS Institute Inc., Cary, NC, USA). The average expression level (T/N ratio) of each miRNA was calculated and log2-transformed. Differences in miRNA expression levels between two variables were analyzed by the Student’s *t* test and logistic regression analysis.

Hierarchical clustering analysis was performed with non-supervised Ward’s method.

The relationship between the expression of miRNAs and various clinicopathological factors were assessed using the chi-squared test. The Kaplan-Meier method was used to estimate patient survival. Differences in postoperative outcome for the expression of the selected miRNAs were analyzed using the log-rank test. *p* < 0.01 was used for significance in microarray analysis with the Student’s *t* test and logistic regression analysis, while *p* < 0.05 was used for significance in the chi-squared test and log-rank test. We constructed receiver operating characteristic (ROC) curves and calculated the area under the curve (AUC) to evaluate the specificity and sensitivity as a postoperative prognostic predictor.

## Results

### Patient information and tumor characteristics

For a training data set, in 36 cases from which we extracted total RNA from archival FFPE samples, we obtained high-quality RNA of both tumors and corresponding normal esophageal epithelia from 19 patients. The clinical information and tumor characteristics of these patients are shown in Table 1. Among the 19 patients, five patients achieved 5-year disease-free survival with an observation period (average ± SD) of 138.7 ± 45.1 months, while 14 patients died of tumor relapse at an observation period (average ± SD) of 14 ± 9.2 months after surgery. These two groups were referred to as the non-relapse group (*n* = 5) and relapse group (*n* = 14). All patients in the relapse group and three of the five patients in the non-relapse group were males. There were no statistical differences between the relapse and non-relapse group in the other clinicopathological characteristics such as age, tumor location, histological grade, pathologic tumor depth, lymph node metastasis, distant metastasis, TNM stage, lymphatic vessel invasion, or venous invasion. All 19 patients underwent radical surgery with no residual tumors (R0). None of the five patients in the non-relapse group received preoperative adjuvant therapy, while three of the 14 (21.4 %) in the relapse group received preoperative chemo- or chemo-radio therapy. One of the five (20.0 %) patients in the non-relapse group and seven of the 14 (50.0 %) patients in the relapse group received postoperative chemo- or chemo-radio therapy. The postoperative observation period (average ± SD) in the non-relapse and relapse groups were 138.7 ± 45.1 and 14 ± 9.2 months, respectively (*p* < 0.001). In the relapse group, the duration between surgery and tumor relapse (average ± SD) was 10.0 ± 8.4 months. The sites of tumor relapse were; liver: 5, lymph node: 2, local recurrence: 1, liver and lymph node: 4, lymph nodes and local recurrence: 2.

**Table 1:** Clinicopathological characteristics of the 19 patients in the training data set

	Non-relapse	Relapse	*p* value
Case number	5	14	
Age (mean ± SD)	68.4 ± 10.7	61 ± 9.6	0.111
Gender			
Male	3	14	0.012
Female	2	0	
Tumor location			
Upper/middle thoracic	1	7	0.244
Lower thoracic	4	7	
Histological grade			
Well/moderate	4	13	0.421
Poorly	1	1	
Pathologic tumor depth			
T1-2	3	3	0.111
T3-4	2	11	
Lymph node metastasis			
N0	2	3	0.418
N1-3	3	11	
Distant metastasis			
M0	5	14	–
M1	0	0	
TNM stage (IUAC TNM Classification 7th edition)			
1–2	3	4	0.211
3–4	2	10	
Lymphatic vessel invasion			
Negative	2	3	0.418
Positive	3	11	
Venous invasion			
Negative	3	4	0.211
Positive	2	10	
Curability			
R0	5	14	–
R1	0	0	
Preoperative chemotherapy	0	2	–
Preoperative chemo radiation therapy (CRT)	0	1	
Postoperative chemotherapy	0	2	–
Postoperative CRT	1	5	
Observation period (month, mean ± SD)	138.7 ± 45.1	14 ± 9.2	<0.001
Duration between surgery and tumor relapse	–	10 ± 8.4	–
Type of tumor relapse			
Liver (H)	0	5	
Lymph node (Ly)	0	2	
Local (L)	0	1	
H + Ly	0	4	
Ly + L	0	2	

For the validation data set, we obtained high-quality RNA from both tumors and corresponding normal esophageal epithelia from 12 ESCC patients. Eight patients were male and four patients were female, and the average age (average ± SD) was 63.6 ± 9.9 years. All 12 patients underwent radical surgery with no residual tumors (R0). Eight out of 12 patients achieved a 5-year disease-free survival with an observation period (average ± SD) of 64.6 ± 18.3 months, while four patients died of tumor relapse at an observation period (average ± SD) of 51.2 ± 27.4 months after surgery (*p* = 0.049, Table 4). These two groups were referred to as the relapse group (*n* = 4) and non-relapse group (*n* = 8).

### The expression of miRNAs in FFPE samples of ESCC tumors detected by microarray

**Fig. 1:**
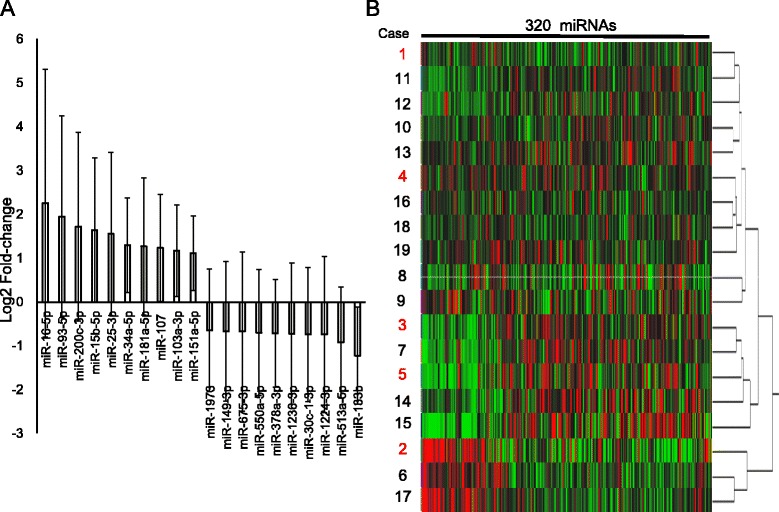
Expression of miRNAs in ESCC. **a** Twenty miRNAs that were expressed in ESCC tumors with more than a twofold difference compared with corresponding normal counter parts. **b** Cluster analysis. The diagram shows the results of the hierarchical clustering of miRNAs and 19 samples. *Columns*: 320 miRNAs that were detected in all 19 cases. *Red* represents a higher expression level; *green* represents a lower expression level. Rows: samples. Cases 1–5 (highlighted in *red*) were non-relapse cases. Cases 6–19 were relapse cases

In the 885 miRNAs assessed using the miRNA oligo chip, the signals of 320 miRNAs were detected in all examined samples. Comparisons between the average expression levels of miRNAs in the 19 ESCC tumors and those in the 19 corresponding normal tissues revealed that the expression of 10 miRNAs (miR-16, miR-93, miR-200c, miR-15b, miR-25-3p, miR-34a, miR-181a, miR-107, miR-103a, and miR-151a) were more than twofold higher, while that of another 10 miRNAs (miR-133b, miR-513, miR-1224, miR-30c, miR-1236, miR-378a, miR-550a, miR-675, miR-149, and miR-1973) were more than twofold lower in the tumors than in their normal counterparts with statistical difference (Fig. 1a). The relationship between the expression of these 20 miRNAs in the tumors (T/N ratio) and clinicopathological features of the tumors were summarized in Additional file 1: Table S1. The higher expression of miR-200c correlated with progressed TNM Stage (*p* = 0.038) and postoperative tumor relapse (*p* = 0.038). On the other hand, the expression of the other 19 miRNAs in tumors did not show correlation with clinicopathological characteristics of the patients. Hierarchical clustering based on all 320 detected miRNAs did not show any relationship between clusters and postoperative tumor relapse (Fig. 1b).

### Differentially expressed miRNAs between patients with and without postoperative tumor relapse

**Fig. 2:**
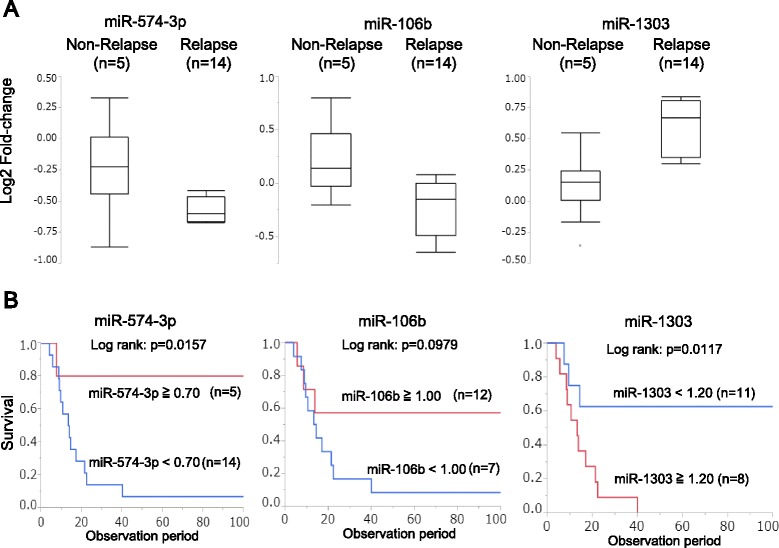
The relationship between the expression of miRNAs (miR-574-3p, miR-106b, and miR-1303) and patient outcome in the training cohort. **a** The expression of miR-574-3p, miR-106b, and miR-1303 in non-relapse and relapse groups. **b** The association between the expression of  miRNAs (miR-574-3p, miR-106b, and miR-1303) and patient prognosis

When the expression (T/N ratio) of miRNAs was compared between relapse (*n* = 14) and non-relapse (*n* = 5) cases using two types of statistical method (*t* test and logistic regression analysis), the lower expression of miR-574-3p and miR-106b, as well as higher expression of miR-1303, correlated with postoperative tumor relapse with statistical significance (*p* < 0.01, Table 2). The Box-and-Whisker plots for these three miRNAs were shown in Fig. 2a.

**Table 2:** Expression of miRNAs associated with postoperative tumor relapse

	Non-Relapse (*n* = 5)		Relapse (*n* = 14)		*t* test	Logistic regression analysis
	Average	SD	Average	SD	*p* value	*p* value
miR-574-3p	0.87	0.18	0.67	0.05	0.001	0.009
miR-106b	1.18	0.26	0.87	0.16	0.004	0.005
miR-1303	1.11	0.17	1.53	0.24	0.007	0.001
miR-1203	1.34	0.70	0.73	0.24	0.006	0.011
miR-1909	0.92	0.17	0.71	0.13	0.009	0.016
miR-204	1.10	0.35	0.76	0.23	0.016	0.033
miR-371-3p	1.27	0.31	0.94	0.25	0.021	0.019
miR-886-3p	0.90	0.49	1.49	0.55	0.037	0.033

In addition, the lower expression of other four miRNAs (miR-1203, miR-1909, miR-204, miR-371-3p), and higher expression of miR-886-3p, correlated with postoperative tumor relapse (*p* < 0.05, Table 2). Based on a ROC curve analysis to differentiate patients with relapse from patients without relapse, the cut-off value, the largest AUC value, sensitivity, and specificity for the expression of the top three miRNAs are shown in Table 3.

**Table 3:** Cut-off value for predicting relapse analyzed using receiver-operating characteristic (ROC) curves

Name	Cut-off	AUC	Sensitivity	Specificity
miR-574-3p	0.70	0.89	0.93	0.80
miR-106b	1.00	0.89	0.79	0.80
miR-1303	1.20	0.94	0.86	1.00

Based on the cut-off value listed in Table 3, the overall survival rate of the patients with high expression of miR-574-3p, low expression of miR-1303 were significantly better (Fig. 2b, d). On the other hand, the expression of miR-106b did not correlate with the overall survival rate of the patients (Fig. 2c).

### The expression of miR-574-3p, miR-106b and miR-1303 in frozen samples of ESCC detected by quantitative RT-PCR analysis

**Fig. 3:**
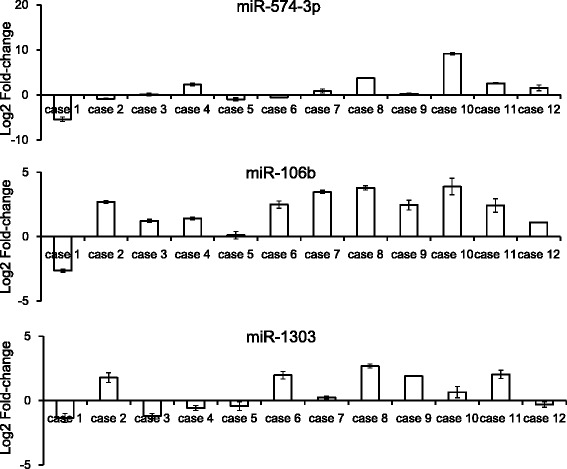
The expression of miRNAs (miR-574-3p, miR-106b and miR-1303) in ESCC patients in the validation cohort. **a** The expression (T/N ratio) of miR-574-3p in frozen samples of surgically removed ESCC specimens detected by RT-PCR. **b** The expression (T/N ratio) of miR-106b in frozen samples of surgically removed ESCC specimens detected by RT-PCR. **c** The expression (T/N ratio) of miR-1303 in frozen samples of surgically removed ESCC specimens detected by RT-PCR

**Fig. 4:**
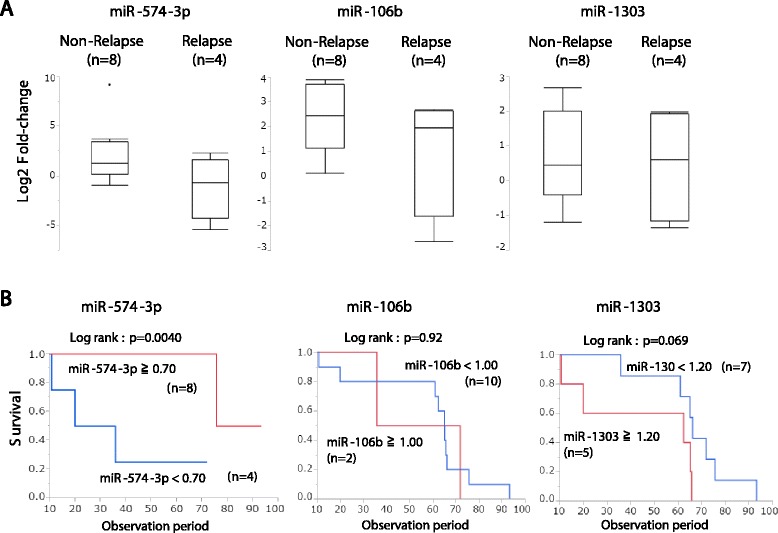
The relationship between the expression of miRNAs (miR-574-3p, miR-106b, and miR-1303) and patient outcome in the validation cohort. **a** The expression of miR-574-3p, miR-106b and miR-1303 in non-relapse and relapse groups. **b** The association between miR-574-3 expression and patient prognosis. **c** The association between miR-106b expression and patient prognosis. **d** The association between miR-1303 expression and patient prognosis

Compared with the corresponding normal esophageal mucosa, the expression of miR-574-3p, miR-106b and miR-1303 were upregulated in 8/12 (66.7 %), 11/12 (91.7 %), and 7/12 (58.3 %) of the validation set patients (Fig. 3a–c). When the expression (T/N ratio) of miRNAs was compared between relapse (*n* = 4) and non-relapse (*n* = 8) cases, higher expression of miR-574-3p was inversely correlated with tumor relapse (*p* = 0.029), while the expression of miR-106b and miR-1303 did not show correlation with tumor relapse (Table 4). The Box-and-Whisker plots for these three miRNAs are shown in Fig. 4a.

**Table 4:** Clinicopathological characteristics of the 12 patients in the validation set

	Non-relapse	Relapse	*p* value
Case number	8	4	
Age (mean ± SD)	63.0 ± 10.3	66.7 ± 8.0	0.425
Gender			
Male	4	4	0.08
Female	4	0	
Tumor location			
Upper/middle thoracic	5	2	0.67
Lower thoracic	3	2	
Histological grade			
Well/moderate	7	2	0.16
Poorly	1	2	
Pathologic tumor depth			
T1-2	2	1	1.00
T3-4	6	3	
Lymph node metastasis			
N0	3	1	0.66
N1-3	5	3	
Distant metastasis			
M0	8	4	-
M1	0	0	
TNM stage			
1–2	3	1	0.66
3–4	5	3	
Lymphatic vessel invasion			
Negative	3	1	0.66
Positive	5	3	
Venous invasion			
Negative	2	1	1.00
Positive	6	3	
Curability			
R0	8	4	–
R1	0	0	
Preoperative chemotherapy	5	3	–
Preoperative CRT	0	0	
Postoperative chemotherapy	2	3	–
Postoperative CRT	0	0	
Observation period (month, mean ± SD)	64.6 ± 18.3	51.1 ± 33.6	0.049
Duration between surgery and tumor relapse	–	8.8 ± 2.9	–
Type of tumor relapse			
Liver (H)	0	2	
Lymph node (Ly)	0	2	
miR-574-3p (<0.7)	1	3	0.029
miR-574-3p (≥0.7)	7	1	
miR-106b (<1.00)	1	1	0.576
miR-106b (≥1.00)	7	3	
miR-1303 (<1.20)	5	2	0.576
miR-1303 (≥1.20)	3	2

The overall survival rate of the patients with high expression of miR-574-3p was significantly better than that of those with low expression (*p* = 0.004), while the expression of miR-106b and miR-1303 did not show correlation with overall survival rate of the patients (Fig. 4b–d).

## Discussion

In the 320 miRNAs assessed using the miRNA oligo chip, the expression of 20 miRNAs were aberrantly expressed in the tumors compared with their normal counterparts, with more than a twofold difference. Among these 20 miRNAs, 13 miRNAs, such as miR-16 [8], miR-93 [9], miR-200c [10], miR-25-3p [11], miR-34a [12], miR-181a [13], miR-107 [14], miR-103a [15], miR-151a [16], miR-149 [17], miR-550a [18], miR-378a [19], and miR-30c [20], have been associated with the malignant potential of esophageal cancer.

In addition, the other seven miRNAs, such as miR-15b-5p [21], miR-1973 [22], miR-675-3p [23], miR-1236-3p [24], miR-1224-3p [25], miR-513a-5p [26], and miR-133b [27], have been associated with the malignant potential of other types of cancer.

These results indicated that the archival FFPE samples were successfully used to identify differentially expressed miRNAs in this study, and suggested the crucial roles of these miRNAs in the development and progression of ESCC.

The expression profiles of miRNAs have been shown to differentiate molecular subtypes in several types of cancer, such as pancreatic cancer [28] and small cell carcinoma of the esophagus [29]. A previous report from our laboratory has demonstrated that hierarchical clustering based on all 410 detected miRNAs showed two discrete clusters in primary small cell carcinoma of the esophagus, and these two clusters were identical to the two patients groups, i.e., patients with and without postoperative tumor relapse [29]. However, in this present study, hierarchical clustering based on all 320 detected miRNAs did not show a relationship between clusters and postoperative tumor relapse, indicating the need to select a specific gene set to predict postoperative outcomes.

All the 15 miRNAs that were significantly expressed differentially between the relapse and non-relapse groups (*p* < 0.05) have been reported to be either oncogenes [18, 29–36], or tumor suppressors [37–42] in various type of tumors.

In the list of miRNAs, the higher expression of miR-574-3p, which showed the most significant inverse association with postoperative tumor relapse (*p* = 0.001), was linked with a significantly better overall survival rate of the patients in the training set.

In the validation set of the patients, higher expression of miR-574-3p was inversely associated with tumor relapse (*p* = 0.029) and a better overall survival rate (*p* = 0.004), further suggesting that the aberrant expression of the miRNAs identified in this study plays key roles in the postoperative outcome of ESCC.

miR-574-3p has been reported to be a tumor suppressor miRNA in various cancers [43]. In gastric cancer, reduced expression of miR-574-3p in tumors and inhibition of cell proliferation, migration, and invasion in miR-574-3p-transfected cancer cells have been reported [37]. In bladder cancer cells, miR-574-3p has been reported to target mesoderm development candidate 1 (MESDC1) miRNA, subsequently inhibiting cell proliferation, migration and invasion ability, and induced cell apoptosis [44]. In prostate cancer, miR-574-3p was reported to regulate “Wnt signaling” to reduce cell proliferation by targeting epidermal growth factor receptor (EGFR) expression [43]. On the other hand, this is the first report of a correlation between the down-regulation of miR-574-3p expression and poor prognosis in patients with ESCC, suggesting the tumor suppressor effects of miR-574-3p in ESCC.

Although molecular targets of miR-574-3p in ESCC have yet to be investigated, the Wnt signaling pathway has been reported to play an important role in progression, metastasis, and invasion in ESCC [45]. Overexpression of EGFR was also associated with aggressive biological behaviors in ESCC [46]. Accordingly, it is possible that miR-574-3p targets the Wnt signaling pathway and/or EGFR to suppress malignant features in ESCC as well. Investigations to reveal the biological role of miR-574-3p, such as transfection of ESCC cell lines with miR-574-3p expression vector to assess the regulation of malignant phenotype, may provide us with the basis of the prognostic significance of miR-574-3p. It may also provide us with molecular targets to develop novel diagnostic and/or therapeutic strategies.

In this study, we obtained formalin-fixed paraffin-embedded (FFPE) samples of the tumors and their normal counterparts, with adequate tissue size from 36 cases and extracted high-quality RNA from 19 patients. After these sample collection with technical limitation, clinicopathological characteristics of the 19 patients summarized in Table 1 showed distinctive features compared to general population of ESCC patients. For example, higher incidence of postoperative tumor relapse (14/19, 73.7 %) was seen and the major type (9/14, 64.3 %) of the tumor relapse were hematologic metastasis.

Therefore, it is possible that the pathological T and N factor did not correlate with postoperative tumor relapse in our study because of the deviation in the sample collection. Further investigations based on the large-scale collection of samples without deviation is needed to confirm our results.

## Conclusions

Our microarray analysis using the archived FFPE samples of surgically removed ESCC tumors from 19 training set patients identified 8 miRNAs that were differentially expressed between the patients with and without postoperative tumor relapse. The higher expression of miR-574-3p, which showed the most significant inverse association with postoperative tumor relapse, was significantly associated with better overall survival. Quantitative real-time PCR experiments using frozen samples of ESCC tumors from 12 independent validation set patients confirmed that the higher expression of miR-574-3p was significantly associated with non-tumor relapse and better overall survival.

Although we only examined a small number of the cases, to the best of our knowledge, this is the first study to show the association between the expression of miR-574-3p and postoperative outcomes in ESCC patients. Further investigations based on the large-scale collection of samples are awaited to assess the clinical use of miR-574-3p as prognostic predictor in patients with ESCC. Molecular biological investigations to define the mechanisms by which miR-574-3p suppresses malignant feature of ESCC using cell lines also provide us with the basis of its prognostic significance and a molecular target to develop novel diagnostic and/or therapeutic strategies.

## Additional file

Additional file 1: Table S1. The relationship between the expression of the 20 miRNAs in the tumors (T/N ratio) and clinicopathological features of the patients. (XLSX 20 kb)

## Abbreviations

ESCC: Esophageal squamous cell carcinoma
miRNAs: MicroRNAs
FFPE: Formalin-fixed paraffin-embedded
ROC: Receiver-operating characteristic
AUC: Area under the curve
